# Novel circulating protein biomarkers for thyroid cancer determined through data-independent acquisition mass spectrometry

**DOI:** 10.7717/peerj.9507

**Published:** 2020-07-06

**Authors:** Dandan Li, Jie Wu, Zhongjuan Liu, Ling Qiu, Yimin Zhang

**Affiliations:** 1 Department of Laboratory Medicine, Peking Union Medical College Hospital, Peking Union Medical College & Chinese Academy of Medical Science, Beijing, China; 2 Department of Clinical Laboratory, Zhejiang Cancer Hospital, Hangzhou, Zhejiang, China

**Keywords:** Thyroid cancer, Proteomics, Complement factor H-related protein 1, Serum, Diagnosis, Biomarker

## Abstract

**Background:**

Distinguishing between different types of thyroid cancers (TC) remains challenging in clinical laboratories. As different tumor types require different clinical interventions, it is necessary to establish new methods for accurate diagnosis of TC.

**Methods:**

Proteomic analysis of the human serum was performed through data-independent acquisition mass spectrometry for 29 patients with TC (stages I–IV): 13 cases of papillary TC (PTC), 10 cases of medullary TC (MTC), and six cases follicular TC (FTC). In addition, 15 patients with benign thyroid nodules (TNs) and 10 healthy controls (HCs) were included in this study. Subsequently, 17 differentially expressed proteins were identified in 291 patients with TC, including 247 with PTC, 38 with MTC, and six with FTC, and 69 patients with benign TNs and 176 with HC, using enzyme-linked immunosorbent assays.

**Results:**

In total, 517 proteins were detected in the serum samples using an Orbitrap Q-Exactive-plus mass spectrometer. The amyloid beta A4 protein, apolipoprotein A-IV, gelsolin, contactin-1, gamma-glutamyl hydrolase, and complement factor H-related protein 1 (CFHR1) were selected for further analysis. The median serum CFHR1 levels were significantly higher in the MTC and FTC groups than in the PTC and control groups (*P* < 0.001). CFHR1 exhibited higher diagnostic performance in distinguishing patients with MTC from those with PTC (*P* < 0.001), with a sensitivity of 100.0%, specificity of 85.08%, area under the curve of 0.93, and detection cut-off of 0.92 ng/mL.

**Conclusion:**

CFHR1 may serve as a novel biomarker to distinguish PTC from MTC with high sensitivity and specificity.

## Introduction

Thyroid cancer (TC) is the eighth most common cancer in women, and it is known to be the most prevalent endocrine malignancy worldwide, with its incidence continuing to increase in recent years (Pellegriti et al., 2013). In particular, after adjusting for age, the incidence of TC was 11 cases per 100,000 individuals in 2006, with a 2.9-fold higher rate of occurrence in women (female:male ratio = 16.3:5.7) (Navas-Carrillo et al., 2014). TCs can be divided into two main groups of neoplasias based on their cell types: one includes carcinomas originating from the follicular epithelium, representing over 95% of all TCs, including papillary TC (PTC, 85%), follicular TC (FTC, 11%), Hurthle cell TC (3%), and anaplastic TC (ATC, 1%), while the other includes medullary TCs (MTCs) originating from the parafollicular thyroid cells, which make up less than 5% of all TCs. Notably, the disparity in TC incidence between the sexes is specific to particular histological subtypes of TC, as there are similar incidences in men and women of the more aggressive types of TC, such as ATC and MTC (Rahbari, Zhang & Kebebew, 2010).

The techniques currently available for use in TC diagnosis include neck ultrasounds, laryngeal examinations, blood tests for thyroid function, chest X-rays, thyroid scans, and fine needle aspiration biopsies (FNAB). FNAB is the most effective technique for MTC or PTC classification. However, the European Thyroid Association Guidelines (Paschke et al., 2017) state that this method is not diagnostic in 2–16% of cases, especially when the amount of aspirated materials tested is inadequate, and cytodiagnostic errors or missed sampling can still occur. In addition, although some traditional TC biomarkers exist for laboratory use, they are not sensitive or specific enough for the differential diagnosis of different TC types. In a postoperative setting, thyroglobulin (Tg) levels are monitored to detect disease recurrence, although this method is limited by the fact that Tg antibodies are common and they interfere with Tg measurements. Tg functions as a useful postoperative marker for well-differentiated TCs, but not for MTCs. Nevertheless, Tg remains the most widely used TC biomarker in clinical settings (Nixon et al., 2017). In addition to MTC, elevated calcitonin (CT) levels may be observed in patients with hypercalcemia, neuroendocrine tumors, renal insufficiency, PTC, FTC, and goiter (Toledo et al., 2009). Furthermore, the presence of heterophilic antibodies along with CT can falsely elevate measured serum CT levels (Preissner et al., 2005).

Therefore, it is necessary to identify novel biomarkers using new methods. Surface-enhanced laser desorption ionization time-of-flight mass spectrometry (SELDI-TOF-MS) (Mato et al., 2015; Paricharttanakul et al., 2016; Wang et al., 2006) is the most commonly used proteomics technique for such purposes. SELDI-TOF-MS has previously been used as a tool to analyze plasma protein profiles as prognostic and diagnostic biomarkers in breast cancer patients. However, the inability of this technique to identify specific proteins and its low sensitivity limits its application. In addition, the use of this technique for large sample sets is precluded by the complex sample preparation steps involved (Cohen et al., 2013).

In this study, an Orbitrap Q-Exactive-plus mass spectrometer was used to screen for differentially expressed proteins in TC patients. The Q-Exactive system provides high-quality, full-scan, and tandem mass spectrometry (MS/MS) data by combining quadrupole ion selection with Orbitrap high-resolution scanning, leading to enhanced performance and operability compared to other types of tandem hybrid MS systems. Data-independent acquisition (DIA) is used as a powerful mass spectrometric technique for protein identification and the quantification of complex samples (Reubsaet, Sweredoski & Moradian, 2019). The reliability and reproducibility of DIA in the development of biomarkers for clinical plasma samples have previously been evaluated (Nigjeh et al., 2017). The substrate used in a proteomic assay may comprise the tissue, serum, or saliva. Previous studies have identified protein biomarkers using tissues or cell lines (Bauer et al., 2017). Sofiadis et al. (2012) applied SELDI-TOF-MS to analyze the differences in protein levels between TC and control tissue samples and revealed that the differences in protein expression profiles between the tissues are not equivalent to those in the serum, which indicated that tissue proteomics are not suitable for use in early diagnosis prior to surgery. In comparison, serum samples are abundant and clinical serum extraction is rapid and convenient. However, few studies investigating serum proteomics in TC have been published (Abdullah et al., 2016), and those that are available have often only analyzed the proteomics of a single TC type (Zhan et al., 2018). As there is a continuing need to classify specific types of TCs to determine the appropriate treatment required, it would be beneficial to develop promising biomarkers for the diagnosis and the classification of different TCs. The aim of this study was to identify novel serum proteomic biomarkers for the diagnosis and classification of TC via DIA to facilitate improved TC management.

## Materials and Methods

### Study design and participants

#### Training study

Participants were recruited from Beijing Peking Union Medical Hospital (PUMCH) and Zhejiang Cancer Hospital between February 2017 and January 2018. All enrolled subjects were separated into three main groups: 10 healthy controls (HCs), 15 patients with benign thyroid nodules (TNs), and 29 patients with TCs. Subjects in the TC group were further divided into three subgroups (13 with PTC, 10 with MTC, and six with FTC), based on the histogenesis and morphology of their tumors.

#### Validation study

Participants were recruited from PUMCH and Zhejiang Cancer Hospital between February and December 2018. All enrolled subjects were divided into three main groups: 176 HCs, 69 patients with TNs, and 291 patients with TCs. Subjects in the TC group were further divided into three subgroups (247 with PTC, 38 with MTC, and six with FTC), based on the histogenesis and morphology of their tumors.

Details regarding the inclusion and exclusion criteria used were provided in a previous study (Li et al., 2019). All blood samples were collected after 9–12 h of overnight fasting before thyroidectomy or a health checkup. The blood specimens were stored at −80 °C after centrifugation until further tests were conducted. Histological classification was conducted according to World Health Organization (WHO) criteria. This study was approved by the Ethics Committee of PUMCH (Chinese Academy of Medical Sciences, ZS-1852), and the subjects from Zhejiang Cancer Hospital provided written informed consent.

### Laboratory measurements

#### Training study

In total, 61 serum samples were analyzed to screen them for differentially expressed proteins using DIA. Four main steps of DIA were carried out as follows: sample preparation, high pH reversed phase fractionation, mass spectrometric acquisition, and mass spectrometric data analysis. After this, due to differences in the batch effect found during pre-processing, five samples from the HC group and two samples from the PTC group were removed from the final data analysis. Finally, 54 samples were included in the DIA analysis. The differentially expressed proteins identified and the details of the experimental methods used are described in Supplemental File 1.

#### Validation study

**Figure 1:**
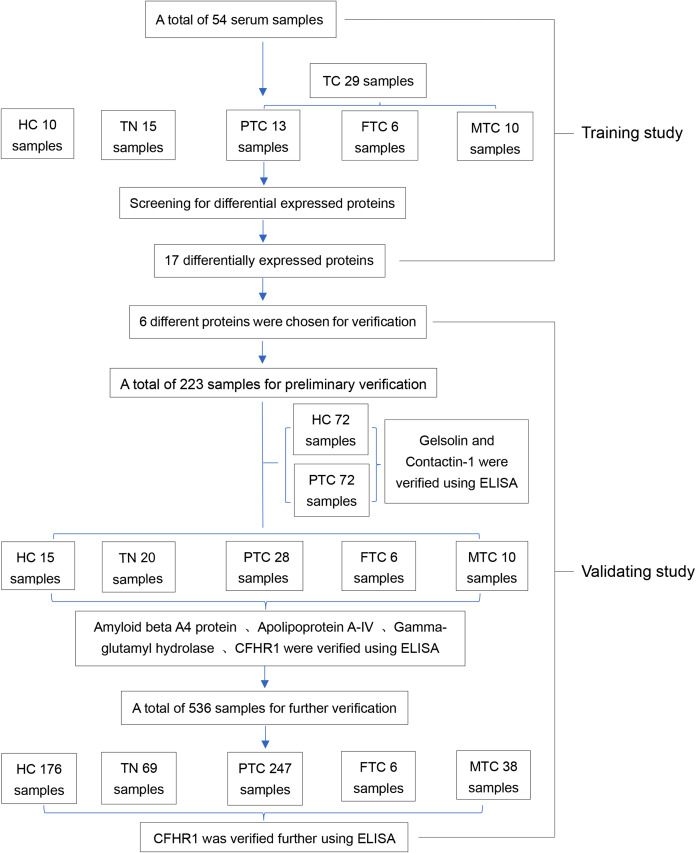
The entire experimental flowchart.

A human complement factor H-related protein 1 (CFHR1) enzyme-linked immunosorbent assay (ELISA) kit (Catalog No: IC-CFHR1-Hu), human contactin 1 (CNTN1) ELISA kit (Catalog No: IC-CNTN1-Hu), human gelsolin ELISA kit (Catalog No: IC-GS-Hu), human amyloid precursor protein ELISA kit (Catalog No: DL-APP-Hu), and human gamma-glutamyl hydrolase ELISA kit (Catalog No: CSB-EL009389HU) were used to validate whether these proteins were differentially expressed in large numbers of samples. Tg and CT levels were measured using a Cobas e601 (Roche, Beijing, China) automatic biochemical analyzer that incorporates Roche Diagnostics reagents. The common internal quality control program standardized by Peking Union Medical College Hospital was applied for the biochemical tests performed in this study. An experimental flowchart of this program is provided in Fig. 1.

### Statistical analyses

The Kolmogorov–Smirnov test was used to estimate the distribution of the data. Normally distributed continuous variables (age) are presented as mean ± standard deviation values and were compared by analysis of variance. Non-normally distributed data are presented as medians (quartiles), and were compared using the Mann–Whitney *U* or Kruskal–Wallis tests. The Bonferroni post hoc test was also used for multiple comparisons. Receiver operating characteristic curves were drawn using MedCalc. Benjamini Hochberg adjustment was used. All *P*-values presented are two-tailed, and values of *P* < 0.05 were considered statistically significant. Analyses were performed using the SPSS 20.0 software (Armonk, NY, USA).

## Results

### Training study

#### Basic information regarding the 54 samples

A total of 54 samples were included in the five groups that were studied. The male to female ratio among the 10 subjects with HC, 15 with TNs, and 13 with PTC was approximately 1:3. The average age of the subjects in the HC group was 45.0 ± 10.23 years, whereas that in the TN group was 49.6 ± 10.38 years and in the PTC group was 41.3 ± 7.81 years. There were no significant differences in age among the three main groups. However, the average ages of patients with FTC (60.2 ± 17.66 years) and MTC (54.1 ± 13.2 years) were significantly different from the ages of patients with TNs, HC, and PTC (*P* < 0.05).

#### Identification of total and differentially expressed proteins

Numerous screened proteins were identified in the TC and non-TC groups. A total of 517 proteins were detected in the serum samples (Table S1). Among the screened total proteins, we selected those with significantly different expression (*P* < 0.05) and with an expression ratio of >2 or <0.5 between the two groups as the differentially expressed proteins for consideration in further analyses. As a result, we designated 17 proteins as differentially expressed proteins (*P* < 0.05 and expression ratio ≥2 or ≤0.5; Table S2). There were eight upregulated (ratio > 2) and nine downregulated proteins (ratio < 0.5) in the TC group compared to those in the non-TC group, with expression ratios (cancer/control) ranging from 0.0433 to 7.799 (Table S3). The results of some screening of the data from the training study were provided in our previous study (Li et al., 2019).

### Validation study

#### Expression levels of the differentially expressed proteins

The amyloid beta A4 protein and apolipoprotein A-IV were the most strongly downregulated proteins in the TC group, with ratios (cancer/control) of 0.0433 and 0.134, respectively. Gamma-glutamyl hydrolase and CFHR1 were the most strongly upregulated proteins in the TC group, with ratios (cancer/control) of 4.669 and 7.799, respectively. Gelsolin and contactin-1 have been reported to be expressed during TC (Kim et al., 2007; Shi et al., 2015), but their role as potential serum protein markers has not yet been studied. Therefore, we selected six proteins (amyloid beta A4 protein, apolipoprotein A-IV, gelsolin, contactin-1, gamma-glutamyl hydrolase, and CFHR1) for further validation in tests with age- and sex-matched samples.

**Figure 2:**
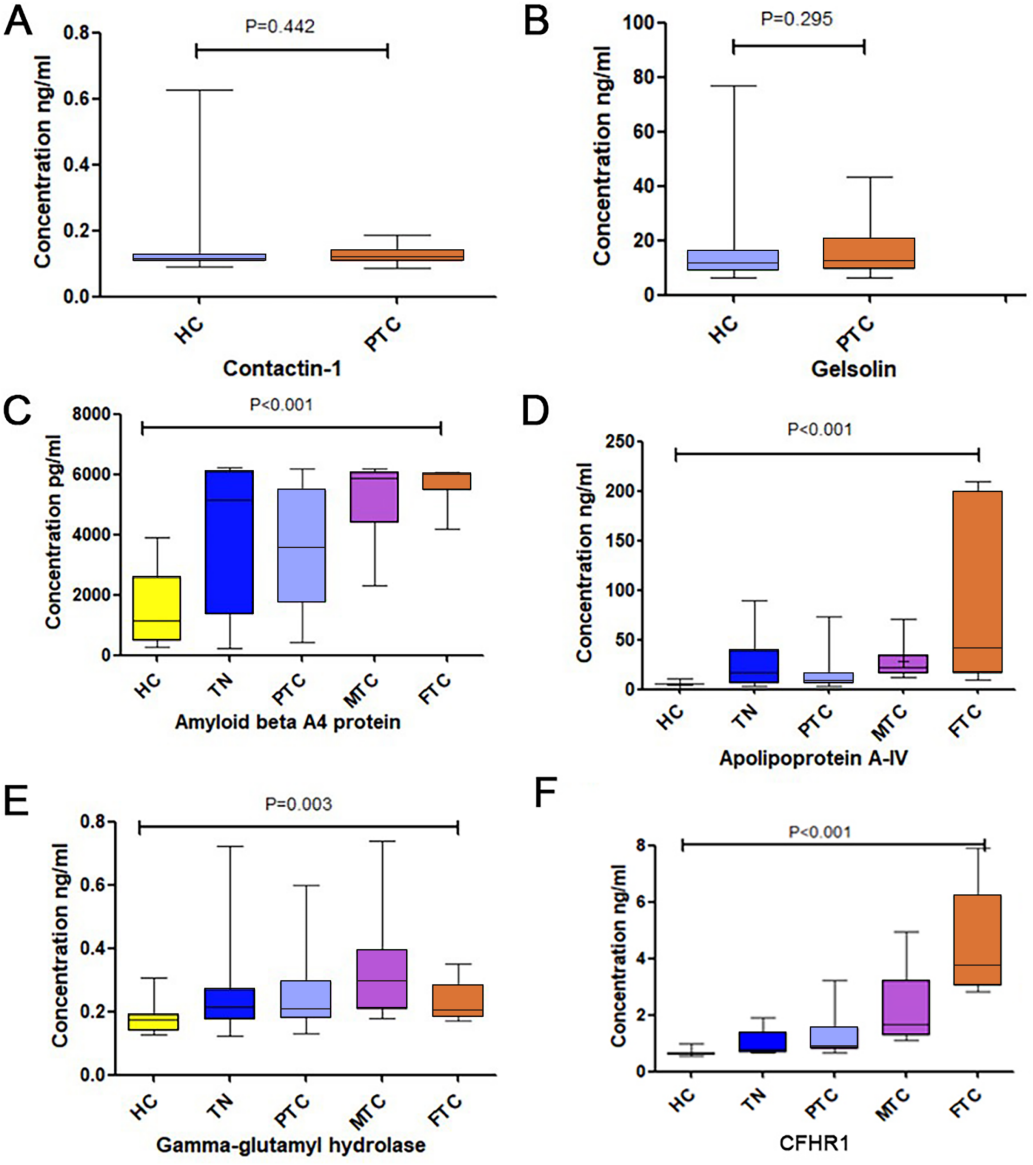
The expression levels of the six chosen differential proteins. (A) The concentration of Contaction-1 between the HC and PTC groups. (B) The concentration of Gelsolin between the HC and PTC groups. (C) The concentration of Amyloid beta A4 protein among the HC, TN, PTC, MTC and FTC groups. (D) The concentration of Apolipoprotein A-IV among the HC, TN, PTC, MTC and FTC groups. (E) The concentration of gamma-glutamyl hydrolase among the HC, TN, PTC, MTC and FTC groups. (F) The concentration of CFHR1 among the HC, TN, PTC, MTC and FTC groups.

The results of tests of these 6 proteins are provided in Table 1, Fig. 2 and Fig. S1. No significant difference was detected in gelsolin and contactin-1 expression levels between HC and PTC, as shown in Figs. 2A and 2B. Although there were significant differences in amyloid beta A4 protein, apolipoprotein A-IV, and gamma-glutamyl hydrolase expression levels among the five groups, the levels of these three biomarkers in patients with TNs were slightly higher than those in patients with PTC, as shown in Figs. 2C–2E. Therefore, we did not test these proteins further.

**Table 1:** The number of patients and the expression of six proteins in the preliminary verification.

Protein	HC Median (Q1–Q3)	TN Median (Q1–Q3)	PTC Median (Q1–Q3)	FTC Median (Q1–Q3)	MTC Median (Q1–Q3)
*N*	87	20	100	6	10
Gelsolin (ng/ml)	11.641 (8.959–15.739)	–	12.561 (9.277–20.396)	–	–
Contactin-1 (ng/ml)	0.143 (0.138–0.152)	–	0.145 (0.138–0.161)	–	–
Amyloid beta A4 protein (ng/ml)	1.136 (0.469–2.599)	5.154 (1.366–6.118)	3.539 (1.755–5.460)	5.978 (5.456–6.046)	5.885 (4.392–6.076)
Apolipo-protein A-IV (ng/ml)	5.331 (4.991–6.086)	17.196 (5.248–38.980)	8.246 (5.412–16.272)	40.214 (15.870–200.095)	21.621 (15.375–34.604)
Gamma-glutamyl hydrolase (ng/ml)	0.176 (0.138–0.190)	0.216 (0.176–0.271)	0.208 (0.177–0.294)	0.203 (0.183–0.284)	0.298 (0.207–0.394)
CFHR1 (ng/ml)	0.635 (0.604–0.683)	0.795 (0.686–1.377)	0.874 (0.789–1.539)	3.757 (3.023–6.217)	1.557 (1.242–3.226)

**Figure 3:**
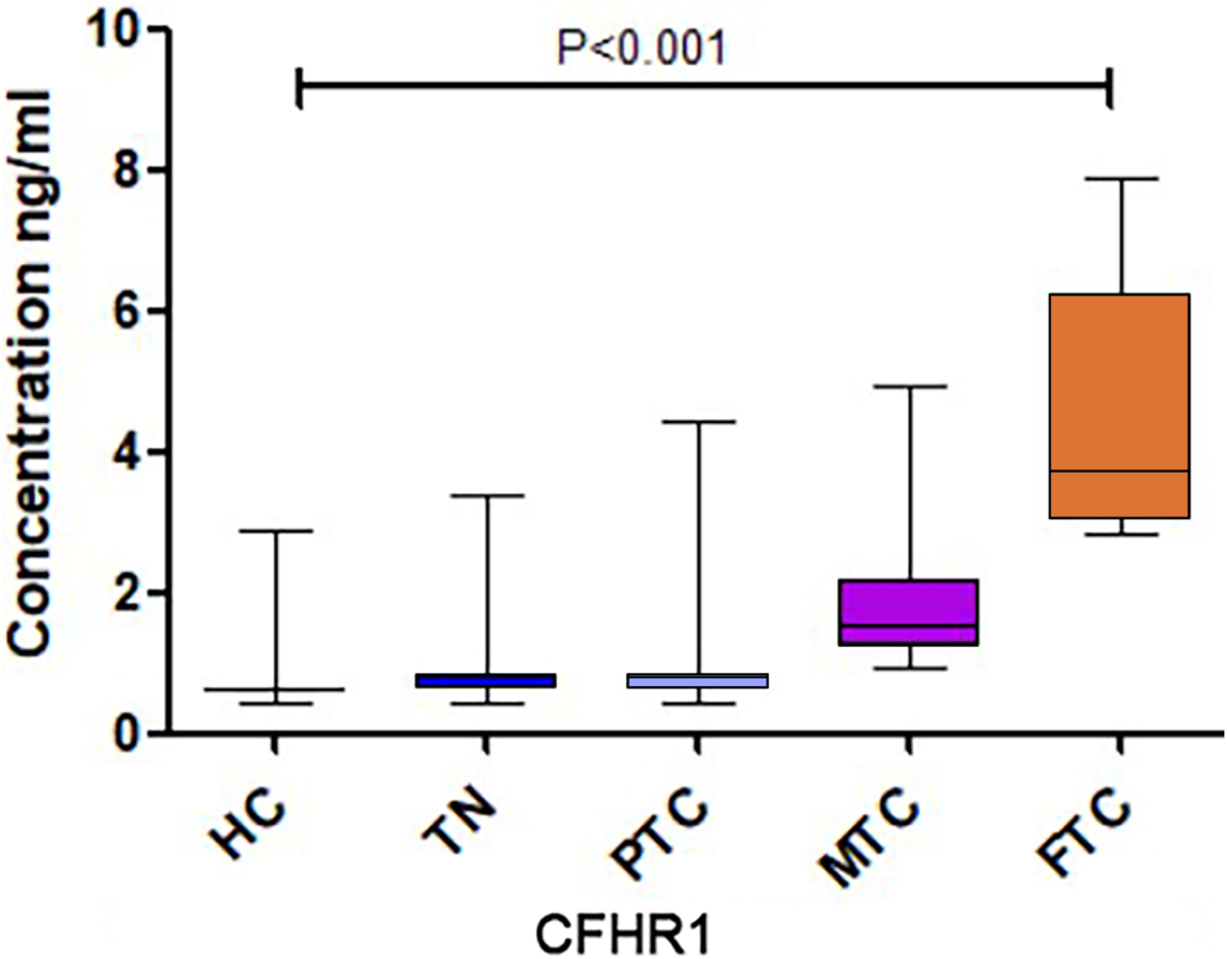
The CFHR1 expression level of further validation.

A significant difference in CFHR1 levels was found among the five groups, and CFHR1 concentrations were higher in the cancer groups compared to that in the control group (Fig. 2F). We increased the sample size to further validate the differences in CFHR1 expression, and the results are shown in Table 2 and Fig. 3. The detailed validation data of CFHR1 are shown in Table S4.

**Table 2:** The number of samples and CFHR1 expression level of further validation.

Protein	HC *N* (median, Q1–Q3)	TN *N* (median, Q1–Q3)	PTC *N* (median, Q1–Q3)	FTC *N* (median, Q1–Q3)	MTC *N* (median, Q1–Q3)
CFHR1 (ng/ml)	178 (0.6272, 0.5626–0.6576)	69 (0.6949, 0.6272–0.8159)	248 (0.7475, 0.6403–0.8267)	6 (3.757, 3.023–6.2168)	38 (1.518, 1.235–2.297)

#### Correlation analysis of CFHR1 levels and clinicopathological characteristics of patients with TC

We further analyzed the association between CFHR1 levels and the clinicopathological characteristics of patients with TC in a validation cohort study. The Mann–Whitney *U* test revealed that there was no statistically significant correlation between CFHR1 levels and metastasis (Table 3). CFHR1 levels did not differ significantly between MTC and FTC at different stages according to the results of the Kruskal–Wallis test. However, CFHR1 levels were significantly different at different stages of PTC (*P* < 0.05), although they did not increase with the development of the disease (i.e., the CFHR1 levels at stage I were higher than those at other stages).

**Table 3:** Association of CFHR1 level in different types of TC with clinicopathological features for enrolled patients.

	PTC *N* (median, Q1–Q3)	MTC *N* (median, Q1–Q3)	FTC *N* (median, Q1–Q3)
Lymphatic metastasis			
Yes	58 (0.7772, 0.6432–0.8270)	14 (2.2162, 1.4985–3.2384)	1 (3.088)*
No	179 (0.7492, 0.6432–0.8287)	5 (1.254, 1.1765–2.93695)	5 (4.274, 3.033–6.774)
*P*-value	0.569	0.257	0.667
Stage			
I	190 (0.7691, 0.6520–0.8340)	2 (2.8645)*	1 (3.24)*
II	13 (0.6009, 0.4717–1.2814)	3 (1.3737)*	3 (4.253)*
III	5 (0.7355, 0.5568–0.8055)	3 (2.6375)*	–
IV	27 (0.7209, 0.5667–0.7884)	11 (2.623, 1.443–3.2322)	2 (5.489)*
*P*-value	<0.05	0.509	0.888

**Note:**

* When *N* < 5, the expression of CFHR1 level is shown in *N* (mean).

#### *Diagnostic performance of CFHR1 among PTC, MTC*, *and FTC*

**Figure 4:**
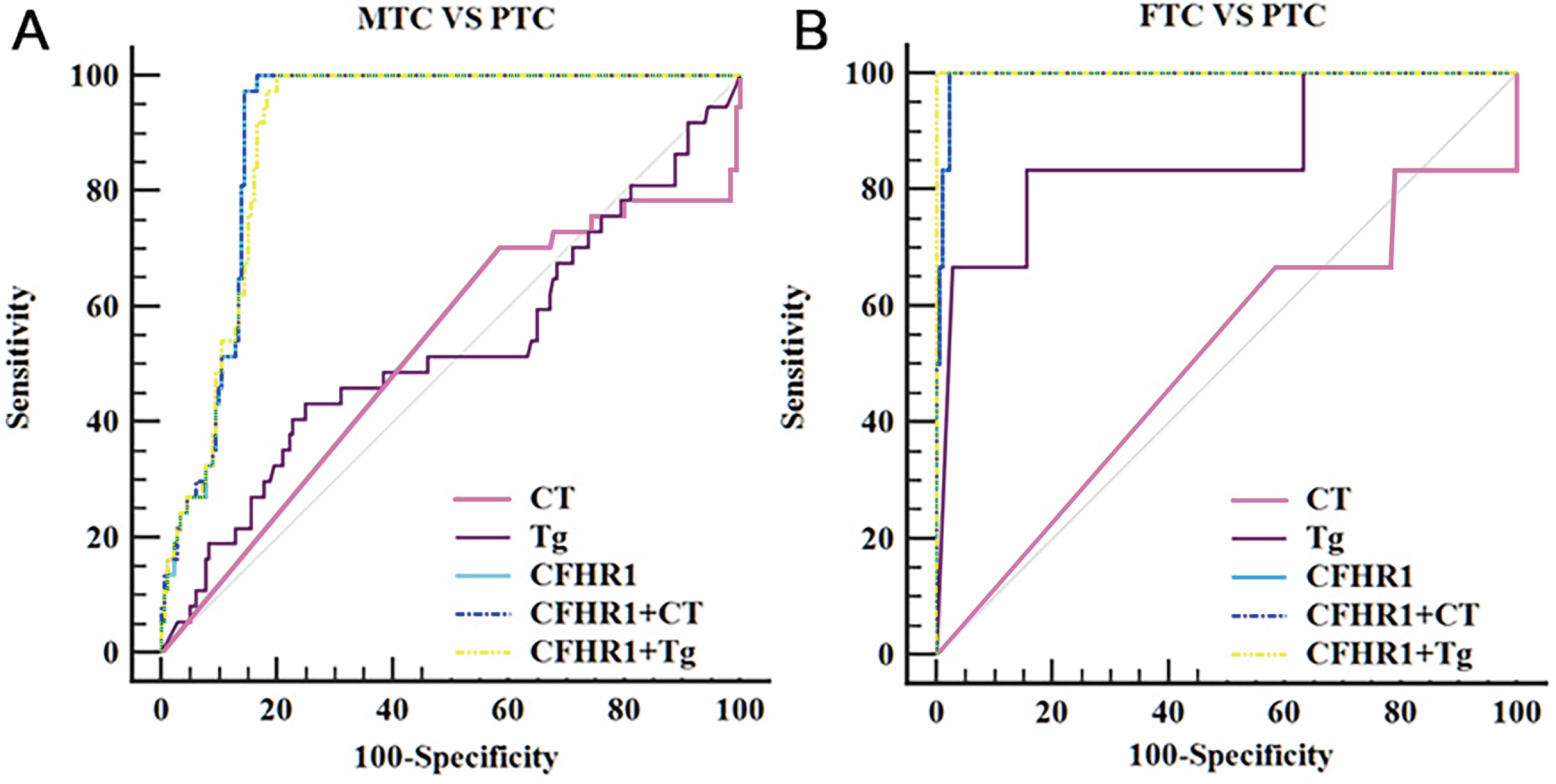
ROC curves of potential biomarker CFHR1 levels for differentiating MTC and FTC from PTC. (A) ROC curves of potential biomarker CFHR1 levels for differentiating MTC from PTC. (B) ROC curves of potential biomarker CFHR1 levels for differentiating FTC from PTC.

Our results revealed that CFHR1 had higher diagnostic performance in distinguishing patients with MTC from those with PTC (*P* < 0.001), as shown in Table 4 and Fig. 4. CFHR1 could distinguish MTC from PTC with a sensitivity of 100.0%, specificity of 85.08%, area under the curve of 0.93, and detection cut-off of 0.92 ng/mL. The combination of CFHR1 and CT increased the specificity to 98.90%. However, the sensitivity and specificity of CFHR1, CT, and Tg, and the combination of CFHR1+CT, to distinguish FTC from PTC were low (*P* > 0.05).

**Table 4:** Diagnostic performance of Tg, CT and CHFR1 for discriminating patients with PTC from PTC and MTC groups.

	Sensitivity % (95% CI)	Specificity % (95% CI)	Cut-off	AUC (95% CI)	*P*-value
MTC vs PTC					
CT	21.62 [9.83–38.21]	98.07 [95.13–99.47]	7.74 pg/ml	0.60 [0.50–0.66]	0.7401
Tg	40.54 [24.75–57.90]	74.80 [66.17–82.19]	21.02 ng/ml	0.58 [0.50–0.65]	0.6459
CFHR1	100.00 [90.75–100.00]	85.08 [80.03–89.27]	0.92 ng/ml	0.93 [0.89–0.95]	<0.001
CFHR1+CT	21.62 [9.83–38.21]	98.90 [96.07–99.87]	–	0.91 [0.87–0.95]	<0.001
FTC vs PTC					
CT	33.33 [4.33–77.72]	69.86 [63.14–75.99]	1.01 pg/ml	0.52 [0.45–0.58]	0.3446
Tg	100.00 [54.07–100.00]	30.08 [22.14–39.00]	5.71 ng/ml	0.65 [0.56–0.73]	0.1074
CFHR1	100.00 [54.07–100.00]	11.69 [7.97–16.36]	0.55 ng/ml	0.56 [0.50–0.62]	0.4496
CFHR1+CT	33.33 [4.33–77.72]	71.58 [64.46–77.99]	–	0.52 [0.45–0.60]	0.3661

**Note:**

CI, confidence interval.

## Discussion

The genetic abnormalities occurring in cancer can produce aberrant protein expression patterns. The recent development of proteomic approaches has allowed disease-specific targets, which can work as promising diagnostic and prognostic biomarkers, to be discovered.

In recent years, the incidence of TC after correction for age and sex has increased by a greater degree than that of all other malignancies, including for men, women, and all ethnic groups (Jemal et al., 2013). However, few good biomarkers, which are essential for the early screening and differential diagnosis of TCs, are currently available. To our knowledge, this was the first study to screen TC serum proteins using the Orbitrap Q-Exactive-plus mass spectrometry system and validate the differential expression of proteins between TC types by ELISA while incorporating a large number of samples.

Among the 17 selected differentially expressed proteins, we selected the 2 with the lowest and highest fold-change (cancer/control), along with gelsolin and contactin-1, which are related to TC, for further validation. Gelsolin is an actin-binding protein that controls actin filament assembly and disassembly (Sun et al., 1999). The expression of gelsolin is frequently reduced in both cancer cell lines and human tumors (Kim et al., 2007). In previous studies, the expression of gelsolin has primarily been determined at the tissue- and cell line-levels (Wang et al., 2017). Aldred et al. (2003) reported that gelsolin expression was downregulated by >2-fold in over 87% of pairwise comparisons of human FTCs with normal thyroid tissues. Kim et al. (2007) evaluated the role of gelsolin in modulating the metastatic potential of FTC. However, few studies have assessed the expression levels of gelsolin in the sera of patients with TC. Our screening results showed that the expression levels of serum gelsolin were downregulated in patients with TC compared to those in the control group. We further verified that no significant difference in gelsolin expression occurred (*P* = 0.295) between PTC and HC, which was likely due to the small number of samples analyzed.

Contactin-1, a member of the immunoglobulin (Ig) family, is a glycosylphosphatidylinositol-anchored neuronal membrane protein (Falk et al., 2002). Recently, several studies have revealed that contactin-1 is an important mediator of the progression of several cancers, including lung adenocarcinoma (Yan et al., 2013), squamous carcinoma (Wu et al., 2012), and hepatocellular carcinoma (Li et al., 2016). However, few studies have explored the role of contactin-1 in TC. Shi et al. (2015) confirmed that knockdown of contactin-1 significantly inhibited tumor proliferation and invasiveness and repressed the expression of cyclin D1 in tissues and cell lines. To our knowledge, no previous study has assessed the expression levels of contactin-1 in the sera of patients with TC. Our screening results showed that the serum expression levels of CNTN1 were upregulated in the TC groups compared to those in the control group. We further verified that this protein occurred at the highest levels in cases of PTC and HC, but no significant differences were found in its expression between these groups (*P* = 0.442).

CFHR1 blocks C5 convertase activity and interferes with C5b surface association to regulate the complement system (Hannan et al., 2016). It competes with complement factor H (CFH) to bind to C3b, thereby functioning as an antagonist of CFH-directed regulation at cell surfaces. Previous studies have also revealed that CFHR1 may play an important role in acute myelogenous leukemia (Fratelli et al., 2016), IgA nephropathy (Zhu et al., 2015), and chronic central serous chorioretinopathy (Schellevis et al., 2018). Arya & Estrela (2018) revealed that CFHR1 can serve as a protein biomarker in urine for the diagnosis of bladder cancer, and Guo et al. (2015) found that CFHR1 levels were significantly higher in Uyghur patients with early-stage cervical carcinomas than in healthy Uyghur women. However, to date, the utility of CFHR1 as a biomarker of TC has not been described. In the screening experiment, we found that CFHR1 exhibited the highest fold-change (cancer/control) among the differentially expressed proteins. Moreover, the CFHR1 levels in each group were verified to show consistent differences with increased sample sizes. Specifically, CFHR1 levels significantly differed between the MTC, FTC, and PTC groups, with CFHR1 levels in MTC and FTC being higher than those in PTC. In comparison, CFHR1 levels did not differ significantly between the PTC and control groups.

We further analyzed the association between CFHR1 levels and the clinicopathological characteristics of patients with TC in a validating cohort study. However, the Mann–Whitney *U* test revealed no statistically significant correlations between CFHR1 levels and metastasis. Instead, our results revealed that CFHR1 exhibited higher diagnostic performance in distinguishing patients with MTC from those with PTC (*P* < 0.001). Although FTC patients presented the highest levels of CFHR1 in this study, the sensitivity and specificity of CFHR1, CT, and Tg, and the combination of CFHR1+CT, to distinguish FTC from PTC were low (*P* > 0.05). This may have occurred because the incidence of FTC was low, and the FTC sample size was therefore small.

The major strengths of the present study were that we analyzed a wide variety of patients with different TC types, screened for differentially expressed proteins in them, compared differences between groups, and validated these with large samples. However, the present study also had several limitations. First, both the screening and validation experiments utilized age- and sex-matching in the HC, TN and PTC groups, but as the incidences of MTC and FTC were low, the sample sizes of these groups were too small for accurate matching to be carried out. However, due to high degree of malignancy of MTC and FTC, the results showed that the CFHR1 levels in these cases were significantly higher than those in cases of PTC. Second, no bioinformatics analysis was performed to analyze the role of novel protein biomarkers in signaling pathways, along with their upstream regulatory molecules and downstream target molecules.

## Conclusion

There still exist no protein biomarkers for the detection of TC with high diagnostic efficiency in clinical practice. Therefore, there is an urgent need to discover new biomarkers for TC and make advancements in detection technologies and accurate assays. The protein CFHR1 may, thus, serve as a potential tumor marker for TC, and the lack of other appropriate biomarkers for TC may compensate for this marker’s lower sensitivity and specificity. However, the number of patients with MTC and FTC in the present study was limited, and further prospective studies are needed to verify our findings. In future studies, we will use proteomics to examine the sera of a larger number of patients with TC and explore the utility of differentially expressed proteins among them as novel blood biomarkers for the differential diagnoses of PTC, MTC, and FTC. Moreover, as little is currently understood regarding the mechanisms involved in CFHR1 function in TC, investigations of the association of CFHR1 protein expression with changes in its gene expression in TC tissues and cells are also warranted.

## Supplemental Information

10.7717/peerj.9507/supp-1 Supplemental Information 1 All identified genes.

10.7717/peerj.9507/supp-2 Supplemental Information 2 The differentially expressed proteins.

10.7717/peerj.9507/supp-3 Supplemental Information 3 The fold-change (cancer/control) of the 17 differentially expressed proteins.

10.7717/peerj.9507/supp-4 Supplemental Information 4 The detailed validation data for CFHR1.

10.7717/peerj.9507/supp-5 Supplemental Information 5 The details of the experimental methods in the training study.

10.7717/peerj.9507/supp-6 Supplemental Information 6 The comparison of the expression levels of all 6 differentially expressed proteins between the HC and PTC groups.

## Additional Information and Declarations

### Data Availability

The following information was supplied regarding data availability: Supplementary data are available in the Supplemental Files.
