# Supplementary material for: Novel circulating protein biomarkers for thyroid cancer determined through data-independent acquisition mass spectrometry
Source: PeerJ. 2020 Jul 6;8:e9507. doi: 10.7717/peerj.9507 (PMC7346861; doi:10.7717/peerj.9507)
Supplement: Supplemental Information 5 [file peerj-08-9507-s005.docx]

**Sample preparation**

ProteoPrep Blue Albumin and IgG Depletion Kit (PROTBA; Sigma-Aldrich; Merck KGaA) was used to deplete the serum immunoglobulin (Ig) and albumin according to manufacturer’s instructions. After depletion, serum protein concentration was determined by Bradford assay using BSA as standard. A total of 40 µg proteins were reduced with 25mM DTT at 60℃ for 30 min, alkylated with 50mM iodoacetamide in the dark for 10 min. After alkylation, the sample was loaded on an ultrafiltration filter (10 kDa cutoff, Sartorius, German) for FASP digestion. Trypsin was added at a ratio of 1:100 (enzyme to protein) at 37 °C for 14-16 h. The samples were spun at 20,000 g at 4°C for 10 min. The peptides were desalted using Ziptip pipette tips containing C18 media (Merck KGaA). After drying, the peptides were resuspended in 0.1 % formic acid.

**High pH reversed phase fractionation**
The digests were further fractionated using high pH reversed phase chromatography. A reverse chromatography column (XBridge® peptide BEH C18; Waters Corp) was used to separate the mixed peptides and a RIGOL L-3000 system (RIGOL, Beijing, China) was utilized. The peptide mixtures were dissolved in 100 μL mobile phase A (2% (v/v) acetonitrile, 98% (v/v) ddH_2_O, pH 10) then centrifuged at 14,000 g for 20 min. Supernatant was loaded into the column and eluted stepwise by injecting mobile phase B (98% (v/v) acetonitrile, 2% (v/v) ddH_2_O, pH 10). The flow rate was set at 1 ml/min. The fractions were eluted and collected (1 min each) using step gradients of mobile phase B and combined into 10 fractions.

**Mass spectrometric acquisition**

Samples was analyzed using a self‐made analytical column (75 μm×100 mm, 3 μm) on an EASY‐nLC1200 connected to a Q Exactive plus mass spectrometer (Thermo Scientific). Peptides were eluted by using a binary solvent system with 99.9% H2O, 0.1% formic acid (phase A) and 80% ACN, 19.9% H2O, 0.1% formic acid (phase B). The following linear gradient was used: 3-8% B in 5 min, 8-28% B in 80 min, 28-38% B in 17 min, 38-100% B in 8 min washed at 100% B for 10 min. The eluent was introduced directly to a Q-Exactive plus mass spectrometer via EASY-Spray ion source. Source ionization parameters were as follows: spray voltage, 2.2 kV; capillary temperature, 320 °C.

**Mass spectrometric data analysis**

The DDA files were searched against the human Swiss-Prot fasta database (state 15.03.2018, 20,240 entries), and the Biognosys iRT peptides fasta database. The DDA spectra were analyzed with the Proteome Discoverer 2.1 analysis software using default settings (Trypsin/P, two missed cleavages). Search criteria included carbamidomethyl of cysteine as a fixed modification, oxidation of methionine and acetyl (protein N terminus) as variable modifications. The initial mass tolerance for the precursor was 15 ppm and for the fragment ions was 20 ppm. Peptides and proteins were filtered by 1% false discovery rate (FDR) and proteins were further filtered with criteria of above 1 unique peptide. The DDA spectrum library was used to create DIA spectral library.

DIA data were analyzed with Spectronaut pulsar, a mass spectrometer vendor independent software from Biognosys. The default settings were used for targeted analysis of DIA data in Spectronaut. In brief, retention time prediction type was set to dynamic iRT (adapted variable iRT extraction width for varying iRT precision during the gradient) and correction factor for windows. Mass calibration was set to local mass calibration. Decoy generation was set to scrambled (no decoy limit). Interference correction on MS2 level was enabled, removing fragments from quantification based on presence of interfering signals but keeping at least three fragments for quantification. The false discovery rate was estimated with the mProphet approach and set to 1% at peptide level. Protein inference, which gave rise to the protein groups, was performed on the principle of parsimony using the ID picker algorithm as implemented in Spectronaut. For the analysis of the DIA‐runs with the spectral library, the RAW files were converted into the Spectronaut file format, then the files were calibrated in the retention time dimension using the global spectral library. Subsequently, the recalibrated files were the used for the targeted data analysis with the spectral library without new recalibration of the retention time dimension.
