# Supplementary figures and images for: Novel circulating protein biomarkers for thyroid cancer determined through data-independent acquisition mass spectrometry
Source: PeerJ. 2020 Jul 6;8:e9507. doi: 10.7717/peerj.9507 (PMC7346861; doi:10.7717/peerj.9507)

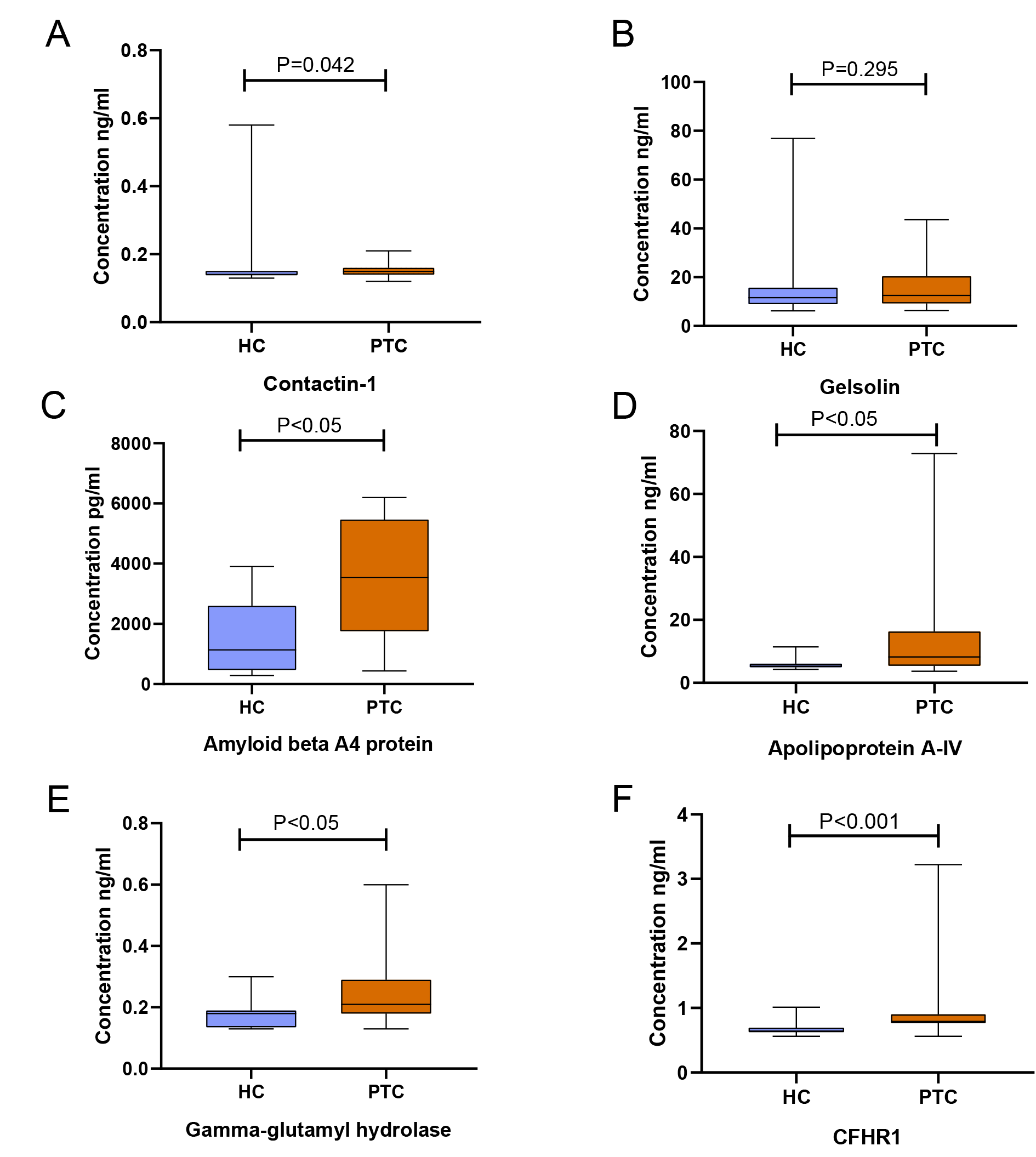

Supplement: Supplemental Information 6 [file peerj-08-9507-s006.png]
